# A Clinical Audit of ECT Documentation in NHS Grampian

**DOI:** 10.1192/bjo.2024.538

**Published:** 2024-08-01

**Authors:** Rachel Ball, Murray Smith

**Affiliations:** NHS Grampian, Aberdeen, United Kingdom

## Abstract

**Aims:**

Electroconvulsive Therapy (ECT) is a treatment used for patients with severe depression, mania, catatonia, and schizophrenia. National Institute for Clinical Excellence (NICE) guidance for the use of ECT advises that for all patients, a risk/benefit assessment for the treatment should be made and documented with particular reference to anaesthetic risk and the adverse effect of cognitive impairment.

For patients who can consent to treatment, NICE recommends the use of patient information leaflets to help people to make an informed decision about their ECT treatment.

For patients who cannot consent to treatment, psychiatrists can authorise the use of ECT using the Mental Health Act. However, NICE recommends that any advance directive should be fully taken into account, and someone who speaks on behalf of the patient should be consulted.

This project aimed to audit whether the documentation of the consent process of patients undergoing ECT in NHS Grampian was in line with the above NICE Guidance.

**Methods:**

The clinical notes and ECT folders of the six patients undergoing ECT treatment in NHS Grampian in January 2023 were reviewed in reference of the following domains:

1. The clinical indication for ECT.
2. If the patient (or their family/advocate) had the opportunity to receive the RCPsych Patient Information Leaflet for ECT.
3. If a discussion about the risks/benefits of ECT had taken place with a patient, their family or advocate.
4. If specific risks and side effects – namely anaesthetic risk and cognitive impairment – had been discussed with the patient, their family or advocate.

The project had been registered with the NHS Grampian Quality Improvement & Assurance Team prior to data collection beginning.

**Results:**

All of the notes reviewed (100%) had the clinical indication for ECT clearly documented.

Three (50%) of the patients had received the RCPsych Patient Information Leaflet for ECT.

A clear risk/benefit assessment discussion was documented in three (50%) of the patients' notes.

Specific discussion of side effects including cognitive impairment and anaesthetic risk was documented in three (50%) of the patients' notes.

**Conclusion:**

There is a clear need for improvement in the documentation of the consent process for ECT in NHS Grampian. While the indication for receiving ECT is being clearly recorded, documentation of the risk/benefit assessment, discussion of specific side effects, and involvement of family or advocacy is less consistent. The introduction of the NHS Grampian standardised consent form is being considered as an option to improve this documentation. The documentation of the consent process for ECT can be re-audited once this form has been introduced.

